# Supplementary material for: Triple-Negative Breast Cancer Subclassified by Immunohistochemistry: Correlation with Clinical and Pathological Outcomes in Patients Receiving Neoadjuvant Chemotherapy
Source: Int J Mol Sci. 2024 May 27;25(11):5825. doi: 10.3390/ijms25115825 (PMC11172922; doi:10.3390/ijms25115825)
Supplement: Supplementary file 1 [file ijms-25-05825-s001.zip › ijms-3001393-supplementary.pdf]

Supplementary S1

Eletronic Case Report Form

DATE OF BIRTH: - -/- -/- - - -
Date of diagnosis: -/- -/- - - -
TNM: Initial clinical stage: [1] I; [2] IIA; [3] IIB; [4] IIIA; [5] IIIB; [6] IIIC
Histological type: [1] IDC; [2] ILC; [3] Others (specified in writing)
Histological grade: [1] Grade 1; [2] Grade 2; [3] Grade 3; [4] Unknown
Ki 67: (specify in writing)%
Neoadjuvant chemotherapy regimen: 1st-line treatment (specified in the text)
2nd line of neoadjuvant treatment: [1] No; [2] RT; [3] CT + RT
Adjuvant chemotherapy regimen: (specify in writing)
Performed surgery: [1] MRM; [2] BS; [3] CC
Pathological response: [1] Complete; [2] Partial; [3] No response
Angiolymphatic invasion: [1] No; [2] Yes; [3] Unknown
Lymphocytic infiltrate: [1] No; [2] Yes; [3] Unknown
Disease recurrence: [1] No; [2] Yes; [3] Unknown
FOXC1 value:
IDO1 value:
E-cadherin value:
AR value:
TNBC subtype:
Date of recurrence: - -/- -/- - - -
Site of recurrence: [1] Visceral; [2] Bone; [3] Soft tissues; [4]
Local first treatment for recurrence: (specify in writing)
Date of last consultation: - -/- -/- - - -
Death: [1] No; [2] Yes; [3] Unknown
Date of death: -/- -/- - - -
Death due to breast cancer: [1] No; [2] Yes; [3] Unknown
Loss to follow-up: [1] No; [2] Yes Date of loss to follow-up: - -/- -/- - - -

Supplementary figures

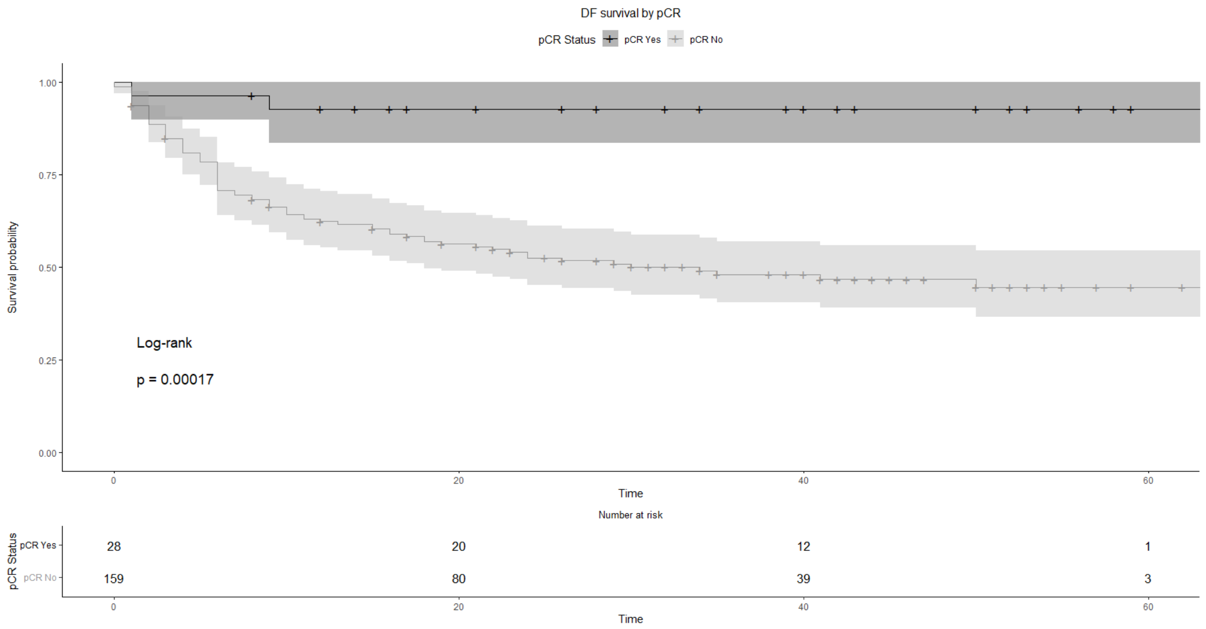

Figure S1. Disease-Free Survival according to pCR status. Legend: DF—disease-free; pCR—pathological complete response. Time is expresed in months.

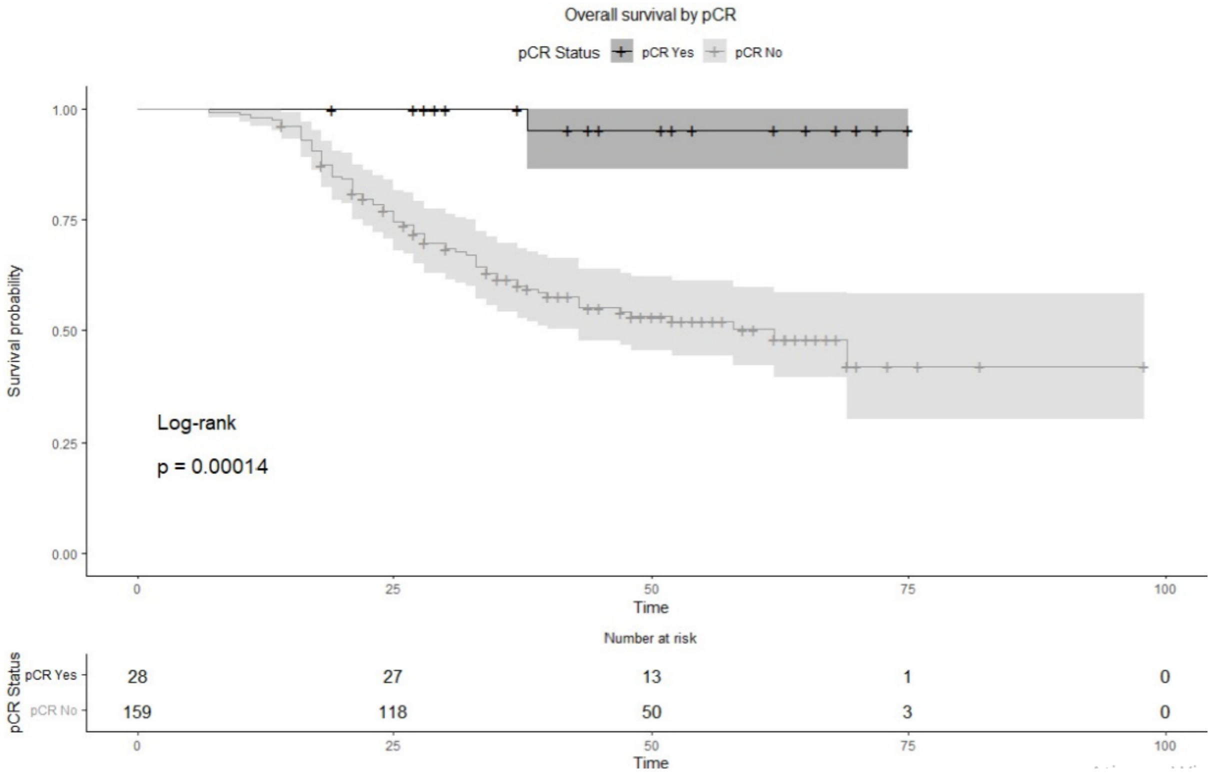

Figure S2. Overall Survival according to pCR status. Legend: pCR—pathological complete response. Time is expresed in months.
